# Vascular Protection of Hydrogen Sulfide on Cerebral Ischemia/Reperfusion Injury in Rats

**DOI:** 10.3389/fneur.2018.00779

**Published:** 2018-10-19

**Authors:** Ji-Yue Wen, Mei Wang, Ya-Nan Li, Hui-Hui Jiang, Xuan-Jun Sun, Zhi-Wu Chen

**Affiliations:** ^1^Department of Pharmacology, Anhui Medical University, Hefei, China; ^2^Department of Pharmacy, Children's Hospital of Soochow University, Suzhou, China

**Keywords:** hydrogen sulfide, ischemia/reperfusion, vascular function, neuronal injury, K_ca_ channel

## Abstract

This study was undertaken to demonstrate the vascular protection of exogenous and endogenous hydrogen sulfide (H_2_S) on cerebral ischemia/reperfusion (I/R) injury. The effect of H_2_S on cerebrovascular dysfunction in middle cerebral artery (MCA) and neuronal damage were measured after cerebral I/R induced by transient middle cerebral artery occlusion (MCAO) in cystathionine c-lyase (CSE) knockdown and wild-type rats. The effect of sodium hydrosulfide (NaHS, donor of exogenous H_2_S), L-cysteine (L-Cys, substrate of endogenous H_2_S), and endothelium cells on the responses of isolated MCA derived from non-ischemic rats was also evaluated to assess the underlying mechanism of H_2_S-mediate cerebral vasodilation. The results revealed that the contraction and dilation of MCA profoundly decreased after cerebral I/R. The vascular dysfunction became more grievous in CSE knockdown rats than in wild-type rats. Interestingly, this vascular dysfunction was significantly alleviated by NaHS supplementation. Moreover, both NaHS and L-cysteine could induce remarkable relaxation in the isolated MCA, which was eliminated by co-application of potassium channel blockers ChTx and Apamin, or endothelial removal. By contrast, adding endothelium cells cultured *in vitro* together with ACh into the luminal perfusate could mimic non-NO and non-PGI_2_ relaxation in endothelium-denuded MCA, once CSE was knocked down from endothelium cells, and its effect on vasorelaxation was abolished. Furthermore, the indexes of neuronal injury were measured after cerebral I/R to confirm the neuroprotection of H_2_S, and we found that the neurological scores, cerebral infarction volume, brain water content, malondialdehyde content, and serum lactate dehydrogenase activity (a marker of cellular membrane integrity) were significantly higher in CSE knockdown rats than in normal control rats. It is not surprising that NaHS could alleviate the cerebral injury. These findings revealed that H_2_S has a protective effect on cerebral I/R injury via its upregulation of the endothelium-dependent contraction and dilation function of cerebral vessels, which may be related to activating potassium channel.

## Introduction

Ischemic stroke is one of the most common cerebrovascular diseases with high mortality and disability rate. Previous studies have shown that ischemia could reduce the cerebral vascular response and change the tension of the vessels, which is the leading cause of disruption of the cerebral blood flow around the ischemic area, and the following hypotension and hypercapnia induced by ischemia could induce vascular dysfunction and finally neuronal injury (1, 2). Autoregulation of cerebral blood vessels is of great importance to protect the neuron against ischemia injury during the hypercapnia and hypotension condition (3, 4). Therefore, the effective treatment for ischemic stroke depends on a functional and patent vasculature, and hence vascular protection is regarded as an important therapeutic approach to reduce stroke damage (5).

Hydrogen sulfide (H_2_S) is regarded as the third endogenous gasotransmitter (6), following carbon monoxide (CO) and nitric oxide (NO). Accumulated evidence indicates that H_2_S plays a much more active and important role against ischemia/reperfusion (I/R) injury, such as kidney I/R injury (7), myocardial I/R injury (8), and cerebral I/R injury (9). Endogenous H_2_S is mainly produced from L-cysteine (L-Cys) in intracytoplasm by cystathionine r-lyase (CSE), cystathionine β-synthase (CBS), and β-mercaptopyruvic acid in mitochondria by 3-mercaptopyruvate sulfurtransferase (3-MST) (10). In the vasculature, the endogenous H_2_S is mainly produced from L-cysteine by CSE in endothelium (11).

H_2_S plays a number of roles in the central nervous system (CNS) under pathological and physiological states such as anti-inflammation, cytoprotection, antiapoptosis, and antioxidation (12–14). In our previous studies, we found that H_2_S mediated the hyperpolarization and dilation of rat cerebral arteries including the MCA and the basilar artery (BA) (15, 16). However, the effect of H_2_S on the cerebrovascular dysfunction after cerebral I/R is still unclear. In addition, we previously also found that intravenous injection with CSE-siRNA and atelocollagen in rats could remarkably knock down the CSE mRNA and protein expression *in vivo* in cerebral vessels and reduce the production of H_2_S. Moreover, we have revealed that NaHS could augment the K_Ca_ current in CBA vascular smooth muscle cells (17). Therefore, we tested the hypothesis in this study, whether H_2_S could attenuate the cerebrovascular dysfunction and the neuronal damage that follows cerebral I/R. Likewise, we followed the same CSE-siRNA- transfection approach to knockdown the CSE expression and reduce the H_2_S production for investigating the effect of endogenous H_2_S on cerebrovascular dysfunction and neuronal damage. In addition, we also sought to explore the role of exogenous H_2_S on cerebral I/R injury and further investigate the underlying mechanism of vascular protection of H_2_S.

## Materials and methods

### Reagents

CSE-siRNA and negative siRNA were purchased from GenePharma (Shanghai, China), and atelocollagen was purchased from KOKEN (Tokyo, Japan); CSE antibody was purchased from Santa Cruz (Delaware Ave, USA); NaHS, Acetylcholine(ACh), bradykinin, 9, 11-dideoxy-11α, 9α-epoxy-methanoprostaglandin F_2α_ (U46619), and Vinpocetine were purchased from Sigma Chemicals (St. Louis, USA); lactate dehydrogenase (LDH) and malondialdehyde (MDA) assay kits were purchased from Nanjing Jiancheng Biological Co (Nanjing, China). ChTx, Apamin, L-Cys, L-NG-nitroarginine methyl ester (L-NAME), and indomethacin (Indo) were purchased from sigma Chemicals (St. Louis, USA); Krebs solution (comprising the following (mM): NaCl 118, KCl 3.4, CaCl_2_ 2.5, KH_2_PO_4_ 1.2, MgSO_4_ 1.2, NaHCO_3_ 25, and glucose 11.1) was aerated with a mixture of 95% O_2_ and 5% CO_2_ and oxygenated during the incubation period.

### Experimental animals

Adult male Sprague-Dawley (SD) rats, weighing between 250 and 300 g, were obtained from the Experimental Animal Center of Anhui Medical University. The animals were allowed free access to water and rodent chow. All experimental procedures were approved by the Ethics Review Committee of Anhui Medical University, which comply with the Guide for the Care and Use of laboratory Animals published by the US National Institutes of Health (NIH publication no. 85-23, revised 2011).

### Cell cultures

Human umbilical vein endothelial cells, EAhy926, were purchased from the Cell Bank, Shanghai Institutes for Biological Sciences, Chinese Academy of Sciences, and were cultured with high glucose Dulbecco's Modified Eagle Medium containing 10% heat-inactivated fetal bovine serum (Gibco), and were transfected by siRNA to knock down the expression of CSE according to the previous research (18).

### CSE-siRNA transfection and cerebral I/R injury model in rats

As described in our previous study (17), the CSE was knocked down with siRNA-transfection technique. The decrease of CSE and its mRNA expression in MCA was used as the indicator of CSE knockdown, measured by western blot and real-time PCR analysis. At 48 h after siRNA-transfection, the cerebral I/R injury of rats was induced by MCAO under chloral hydrate anesthesia (350 mg/kg, ip) (19). Briefly, a 4-0 nylon monofilament suture (total length: 30 mm; diameter: 0.23 mm) was dipped in melted hard wax at the head end, slightly inserted into the right common carotid artery, and pushed ~18–22 mm from the carotid bifurcation to the internal carotid; blood flow of MCA was then blocked at the origin. After 2 h of ischemia, the suture was carefully withdrawn for reperfusion for 24 h. Rats of the non-CSE-siRNA transfected experiment were grouped as: (1) Sham (*n* = 10); (2) MCAO (*n* = 10); (3) MCAO+1 × 10^−5^ mol/kg NaHS (*n* = 10); (4) MCAO+1 × 10^−6^ mol/kg NaHS (*n* = 10); (5) MCAO+1 × 10^−7^ mol/kg NaHS (*n* = 10). Sham group animals were also subjected to the above procedures, except for suture insertion. Rats of CSE-siRNA transfected experiment were grouped as: (1) Sham (*n* = 10); (2) Control (*n* = 10); (3) CSE-siRNA (*n* = 10); (4) CSE-siRNA+NaHS (*n* = 10). Sham group animals were also subjected to the above procedures, except for suture insertion. NaHS was injected into the tail vein of rats after ischemia, while the sham and control rats were injected with saline.

### Cerebral vessel experiment

As described previously (17), the brains of MCAO or non-ischemic rats were rapidly removed after sacrifice under anesthesia and placed in precooled Krebs solution. MCA was carefully isolated immediately and cut into serial segments of 3 mm in length. Subsequently, both ends of the vessel segment were cannulated with glass micropipettes, secured with a nylon monofilament suture and then placed in a perfusion chamber. Thereafter, the segments were equilibrated with 37°C Krebs solutions and continuously aerated with a gas mixture of 95% O_2_ and 5% CO_2_ and then pressurized to 85 mmHg. The luminal flow was then adjusted to 150 μl/min. After 60 min of equilibrium, 1 × 10^−7^ mol/l U46619 or 30 mmol/L KCl was added to the luminal perfusate until a stable contraction was obtained. The diameter of the artery of non-ischemic rats was continually measured utilizing E-rule software, and MCA tension of MCAO rats was measured by myograph (17). The percentage of maximum diameter (% Dmax) was calculated and used to evaluate the vascular dilation of non-ischemic rats using the following formula: Dilation (%) = (D_x_ – D_min_)/(D_max_ – D_min_) × 100%, where D_x_ is the diameter after administration of NaHS, L-Cys, or endothelial cells, D_min_ is the stable diameter of artery precontracted with U46619 or KCl, and D_max_ is the initial diameter. The maximum rate of vascular dilation of MCAO rats was calculated using the following formula: Dilation (%) = (T_min_-T_x_)/(T_min_ – T_max_) × 100%, where T_min_ is the stably tension of artery precontracted with U46619, T_x_ is the vascular tension after administration of ACh or vinpocetine, and T_max_ is the initially vascular tension.

### Evaluation of neurological score

Neurological score (20) of rats was evaluated at 24 h after reperfusion. It was scored on a five-point scale: (1) score 0: no neurologic deficit; (2) score 1: a mild focal neurologic deficit (failure to extend left forepaw fully); (3) score 2: a moderate focal neurologic deficit (circling to the left); (4) score 3: a severe focal deficit (falling to the left); (5) score 4: rat could not walk spontaneously and had a depressed level of consciousness.

### Determination of infarction volume and brain water content

At the end of the neurological score test, the rats were sacrificed with anesthesia. The brains were rapidly removed and sliced coronally at a 2 mm interval. The brain slices were then incubated in the dark in 2% TTC in phosphate-buffered solution (PBS) at 37°C for 30 min for staining. Subsequently, the stained slices were placed in 4% paraformaldehyde for 10 min. All the stained brain slices were photographed subsequently to delineate the area of infarct size using Image J, version 1.6 (National Institutes of Health, Bethesda, MD, USA). As described previously (9), the percentage of infarction volume was determined by normalizing the whole brain.

The dry-wet approach was used to measure the brain water content (21). In short, the fresh slices of each brain were weighed to attain the wet weight. The fresh tissues were then dried in an oven at 105°C for 48 h and weighed again to obtain the dry weight. Brain water content was calculated using the following formula:

Brain water content (%) = (wet weight – dry weight)/wet weight × 100%.

### Measurement of serum LDH activity and MDA level

Briefly, serum and supernatant of brain tissue homogenate of rats were collected and transferred to 96 well plates for LDH activity and MDA level analysis, using the biochemistry assay kit (Jiancheng Bioengineering Ltd, Nanjing, China) and abiding by the manufacturer's manual.

### Statistical analysis

Statistical analysis was performed by one-way analysis of variance (ANOVA) followed by the Duncan test to determine the difference between groups. Blood vessel data is presented as mean ± SD, and the other data are expressed as mean ± SEM. The *p* < 0.05 are considered significant.

## Results

### Effect of H_2_S on cerebrovascular function of rats

#### Exogenous H_2_S attenuated cerebrovascular dysfunction induced by cerebral I/R

**Figure 1:**
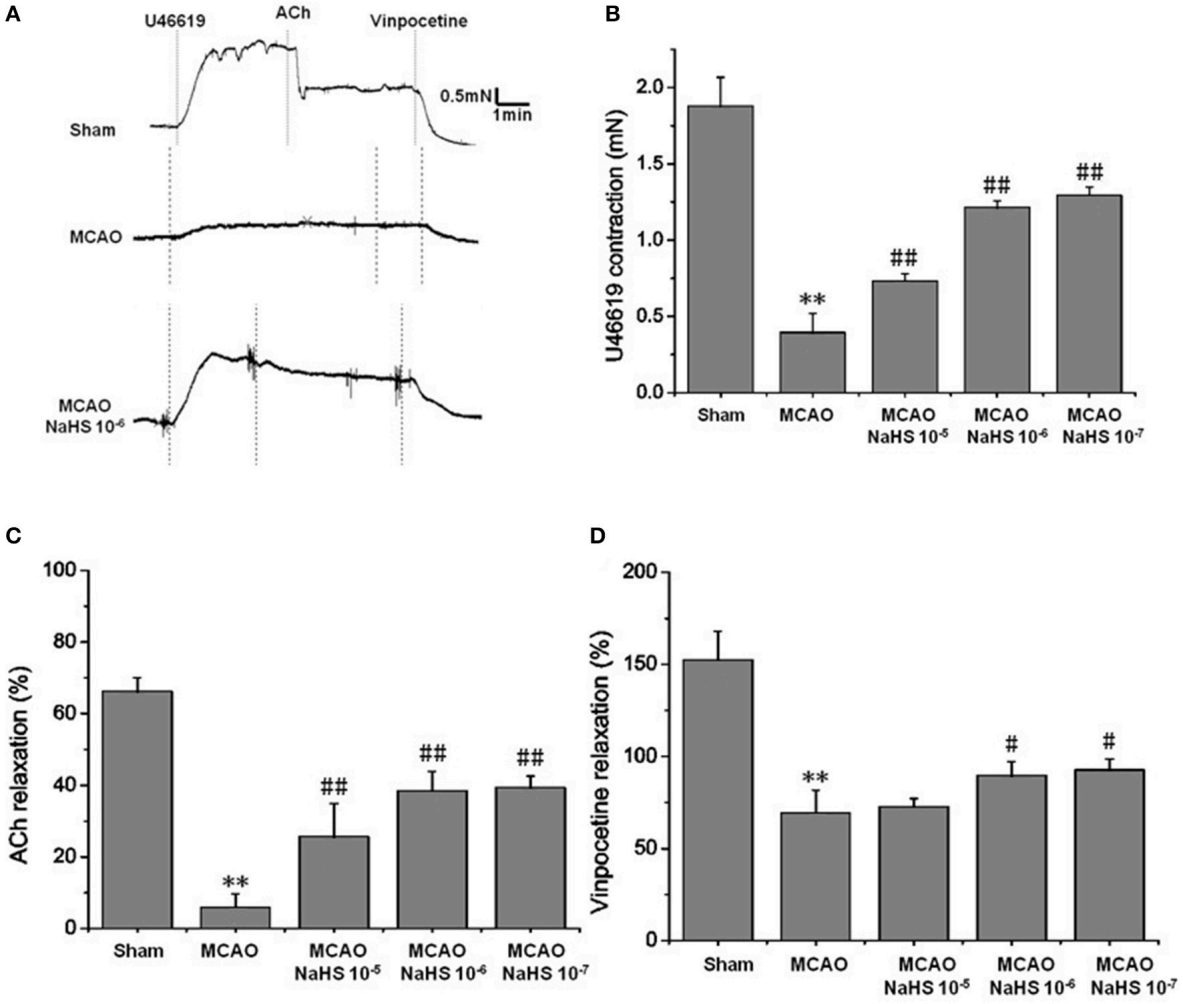
Exogenous H_2_S attenuated cerebrovascular dysfunction induced by cerebral I/R in rats (mean ± SD, *n* = 8). **(A)** Original tracings of U46619-, ACh-, vinpocetine- and NaHS-induced responses of MCA derived from MCAO rats. **(B)** The effect of NaHS on U46619 meditated MCA contraction. **(C)** The effect of NaHS on ACh-meditated relaxation in MCA preconstricted with U46619. **(D)** The effect of NaHS on vinpocetine-meditated relaxation in MCA preconstricted with U46619. ^**^P < 0.01 vs. sham, ^##^*P* < 0.01 vs. MCAO, ^#^*P* < 0.05 vs. MCAO.

Changes of vascular tension in MCA from MCAO rats were examined after cerebral I/R. As shown in Figure 1, in the sham group, 1 × 10^−7^ mmol/L U46619 evoked significant constriction in MCA with maximum response (E_max_) of 1.88 ± 0.19 mN. The contraction in MCA from MCAO rats to U46619 was profoundly decreased and the E_max_ was reduced to 0.39 ± 0.12 mN, but the reduction was significantly ameliorated by 1 × 10^−5^ ~ 1 × 10^−7^ mol/kg NaHS supplement. Furthermore, the ACh-mediated relaxations in MCA were also remarkably inhibited by MCAO, with the E_max_ being reduced from 68.27 ± 3.71% of the sham group rats to 8.91 ± 3.66% of the model group rats, and the dilation dysfunction in MCA was also attenuated by 1 × 10^−5^ ~ 1 × 10^−7^ mol/kg NaHS supplement. In addition, vinpocetine-mediated non-endothelium-dependent relaxation in MCA was also significantly attenuated in the model group (E_max_: 68.44 ± 12.21% in the model group and 151.48 ± 25.32% in the sham group). Interestingly, the decrease of vascular relaxation to vinpocetine injured by cerebral I/R was similarly ameliorated by 1 × 10^−6^ ~ 1 × 10^−7^ mol/kg NaHS supplement. These results indicated that exogenous H_2_S has a protective effect on the cerebrovascular dysfunction injured by cerebral I/R.

### Effect of endogenous H_2_S on cerebrovascular dysfunction injured by cerebral I/R

**Figure 2:**
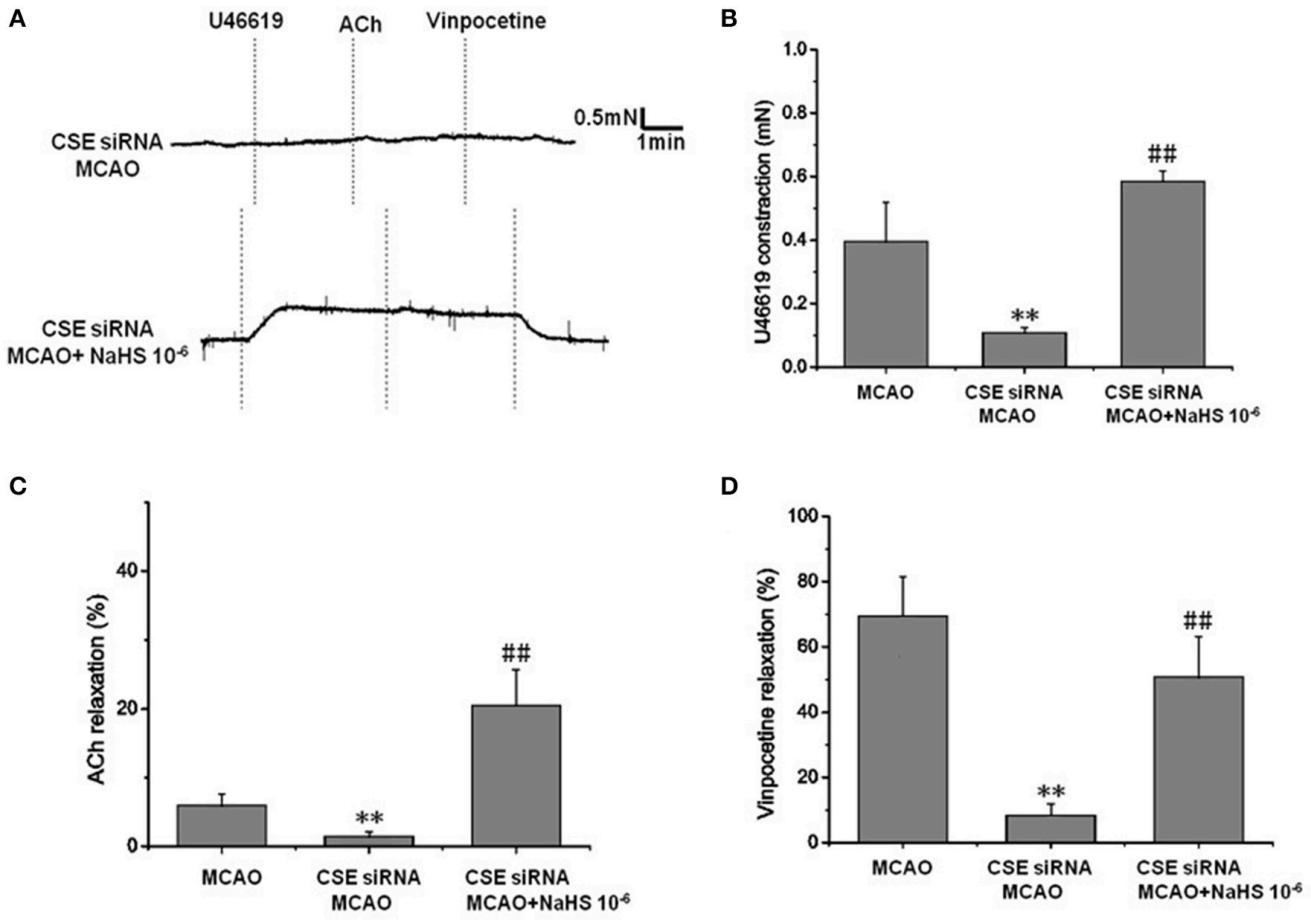
Effect of endogenous H_2_S on cerebrovascular dysfunction injured by cerebral I/R (mean ± SD, *n* = 8). **(A)** Original tracings of U46619-, ACh-, vinpocetine-, and NaHS-induced responses of MCA derived from CSE knocked down rats after MCAO. **(B)** The effect of CSE knockdown and NaHS on U46619-meditated contraction. **(C)** The effect of CSE knockdown on and NaHS- or ACh-meditated relaxation. **(D)** The effect of CSE knockdown and NaHS on vinpocetine-meditated relaxation. ^**^P < 0.01 vs. sham, ^##^*P* < 0.01 vs. MCAO.

In order to clarify the effect of endogenous H_2_S on cerebrovascular dysfunction injured by cerebral I/R, we examined the changes of vascular tension in MCA from CSE knocked down rats after cerebral I/R. As shown in Figure 2, MCA almost had no contractile response to U-46619 (E_max_: 0.11 ± 0.01 mN) in CSE knock down rats after cerebral I/R, which could be significantly elevated by 1 × 10^−6^ mol/kg NaHS supplement (E_max_: 0.59 ± 0.03 mN). Similarly, CSE knockdown attenuated both ACh- and vinpocetine-mediated relaxation of MCA from MCAO rats. E_max_ of ACh-mediated relaxation was reduced from 8.91 ± 3.66% in the MCAO group of wild-type rats to 2.48 ± 2.65% in the CSE-siRNA group; E_max_ of vinpocetine-mediated relaxation was reduced from 68.44 ± 12.21% in the MCAO group of wild-type rats to 8.42 ± 3.47% in the CSE-siRNA group. Interestingly, supplementing with NaHS could also further elevate the ACh- and vinpocetine-mediated vascular relaxation of MCAO rats. These results indicate that the CSE knockdown could induce significant vascular dysfunction, which can be ameliorated by exogenous H_2_S.

### Effect of Ca^2+^-activated K^+^ (K_Ca_) channel blockers on H_2_S-mediated relaxation of MCA

**Figure 3:**
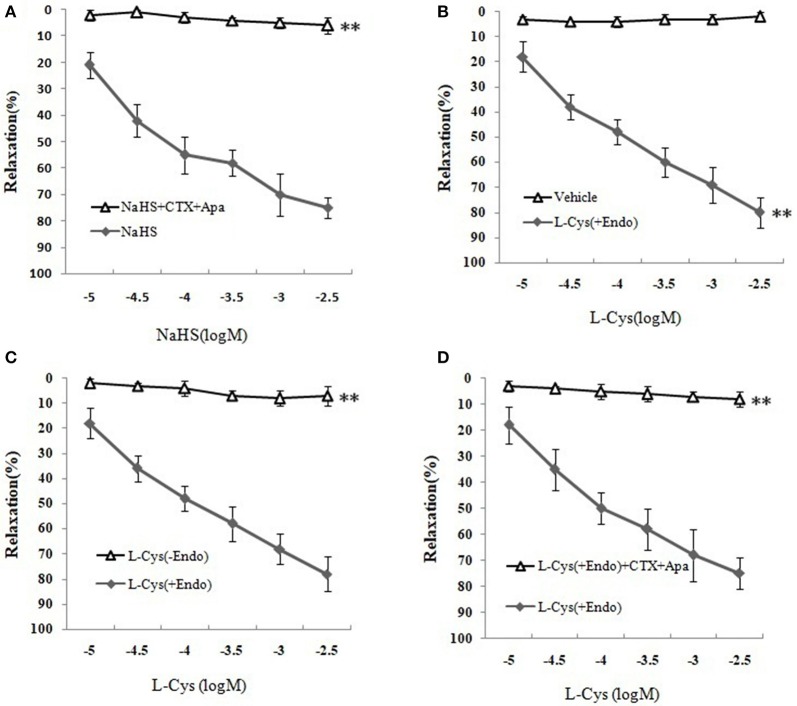
Effects of K_Ca_ channel blockers CTX and Apa on H_2_S-mediated relaxation in MCA of normal rats (mean ± SD, *n* = 8). **(A)** Effects of CTX and Apa on NaHS-induced relaxation. **(B)** Effect of L-Cys on relaxation in U46619-preconstricted rat MCA. **(C)** Effects of the endothelium removal on the L-Cys- induced relaxation. ^**^P < 0.01 vs. L-Cys (+Endo). **(D)** Effect of CTX and Apa on L-Cys-induced relaxation in MCA. ^**^P < 0.01 vs. NaHS or vehicle or L-Cys (+Endo).

We next sought to demonstrate further the effect of H_2_S on MCA and explore the underlying mechanism using K_Ca_ channel blockers CTX and Apa. The results of changes of vascular diameter as shown in Figure 3A and Table 1, the NaHS could induce concentration-dependent dilation in MCA precontracted with U46619 from non-ischemic rats, which was obviously abolished by co-application of CTX and Apa, E_max_ of vascular relaxation being reduced from 76.23 ± 7.4 to 8.75 ± 1.7% after co-application of CTX and Apa. Moreover, L-Cys, the substrate of endogenous H_2_S-producing enzyme similarly induced a concentration-dependent dilation in MCA precontracted with U46619 (Figure 3B, E_max_: 79.28 ± 5.4%, *p* < 0.01 vs. the vehicle group). However, the relaxation in MCA to L-Cys was also obviously abolished by co-application of CTX and Apa, E_max_ being reduced from 79.3 ± 5.4 to 9.7 ± 2.0% (Figure 3D). These data suggested that K_Ca_ channel might be involved in the H_2_S-induced cerebrovascular relaxation.

**Table 1:** Effects of CTX plus Apa on relaxation of MCA to NaHS or L-Cys, and role of vascular endothelium in L-Cys-induced relaxation in the MCA (Mean ± SD, *n* = 8).

**Group**	**Maximum possible effect (%)**
Vehicle	8.03 ± 1.1
NaHS	76.23 ± 7.4^**^
NaHS+ CTX+Apa	8.75 ± 1.7^*^
L-Cys(+Endo)	79.28 ± 5.4^**^
L-Cys(-Endo)	8.85 ± 3.8^##^
L-Cys +Apa +CTX	9.7 ± 2.0^##^

** P < 0.01 vs. vehicle;

* P < 0.01 vs. NaHS;

## *P < 0.01 vs. L-Cys (+Endo)*.

### Effect of vascular endothelium on H_2_S-mediated relaxation of MCA

**Figure 4:**
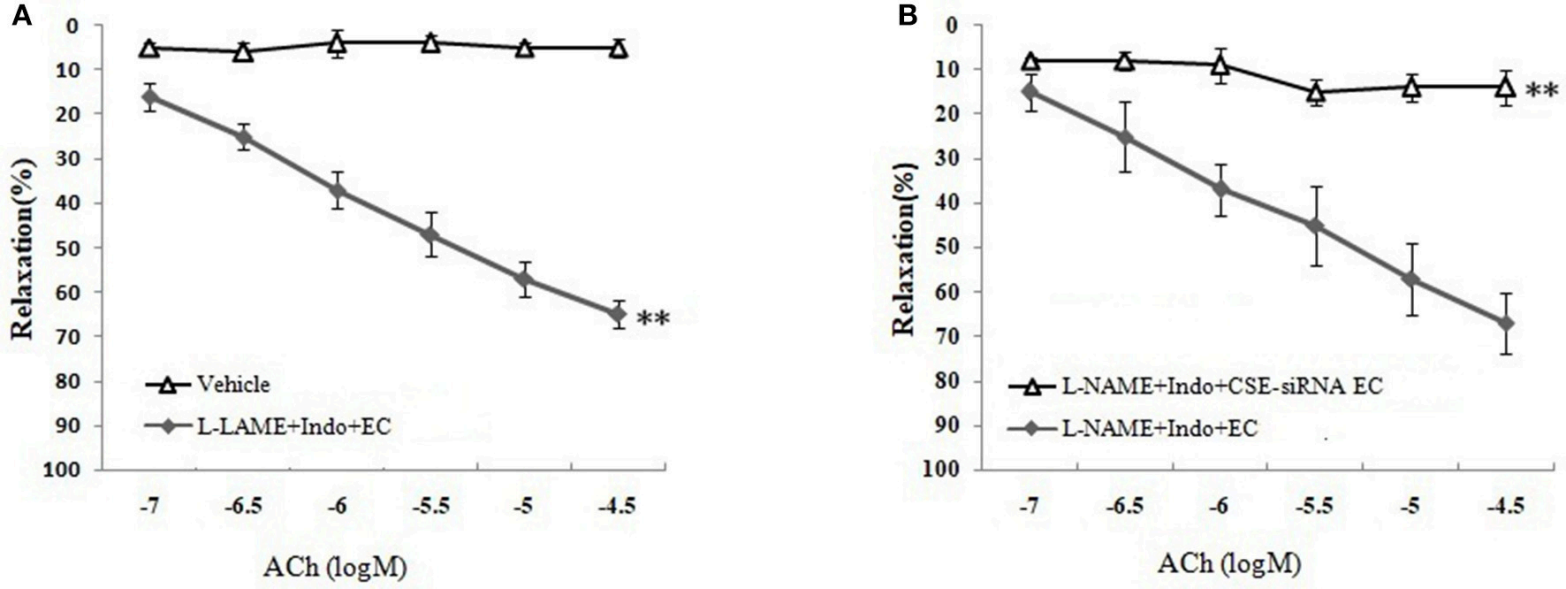
Role of endothelial CSE in ACh-induced non-NO- non-PGI_2_ relaxation in endothelium-denuded rat MCA (mean ± SD, *n* = 8). **(A)** Effect of EAhy926 cells (endothelial cell, EC) on ACh-induced non-NO- non-PGI_2_ relaxation in KCl-preconstricted endothelium-denuded rat MCA. **(B)** Effect of EAhy926 cells with CSE knockdown (CSE-siRNA EC) on ACh-induced non-NO- non-PGI_2_ relaxation in KCl-preconstricted endothelium-denuded rat MCA. ^**^P < 0.01 vs. vehicle or L-NAME+Indo+EC.

As shown in Figures 3B,C and Table 1, the removal of vascular endothelium significantly reduced the relaxation of MCA to L-Cys, with E_max_ being reduced to 8.8 ± 3.8%. Co-adding of ACh and endothelium cells (EAhy926 cells) cultured *in vitro* into luminal perfusate could induce a non-NO and non-PGI_2_ relaxation in endothelium-denuded rat MCA precontracted with KCl (Figure 4, E_max_: 66.1 ± 1.6%). However, co-application of ACh and EAhy926 cells of CSE knockdown cannot induce the relaxation in the endothelium-denuded MCA. These results further suggest that vascular endothelium participated in the relaxation in rat MCA, and that endothelial H_2_S might mediate vasodilation in the blood vessel.

### Effects of H_2_S on neuronal injury induced by MCAO in rats

The rats were transfected with siRNA to knock down the expression of CSE and used to investigate the role of endogenous H_2_S on neuronal injury induced by MCAO.

### Effect of H_2_S on neurological score

**Figure 5:**
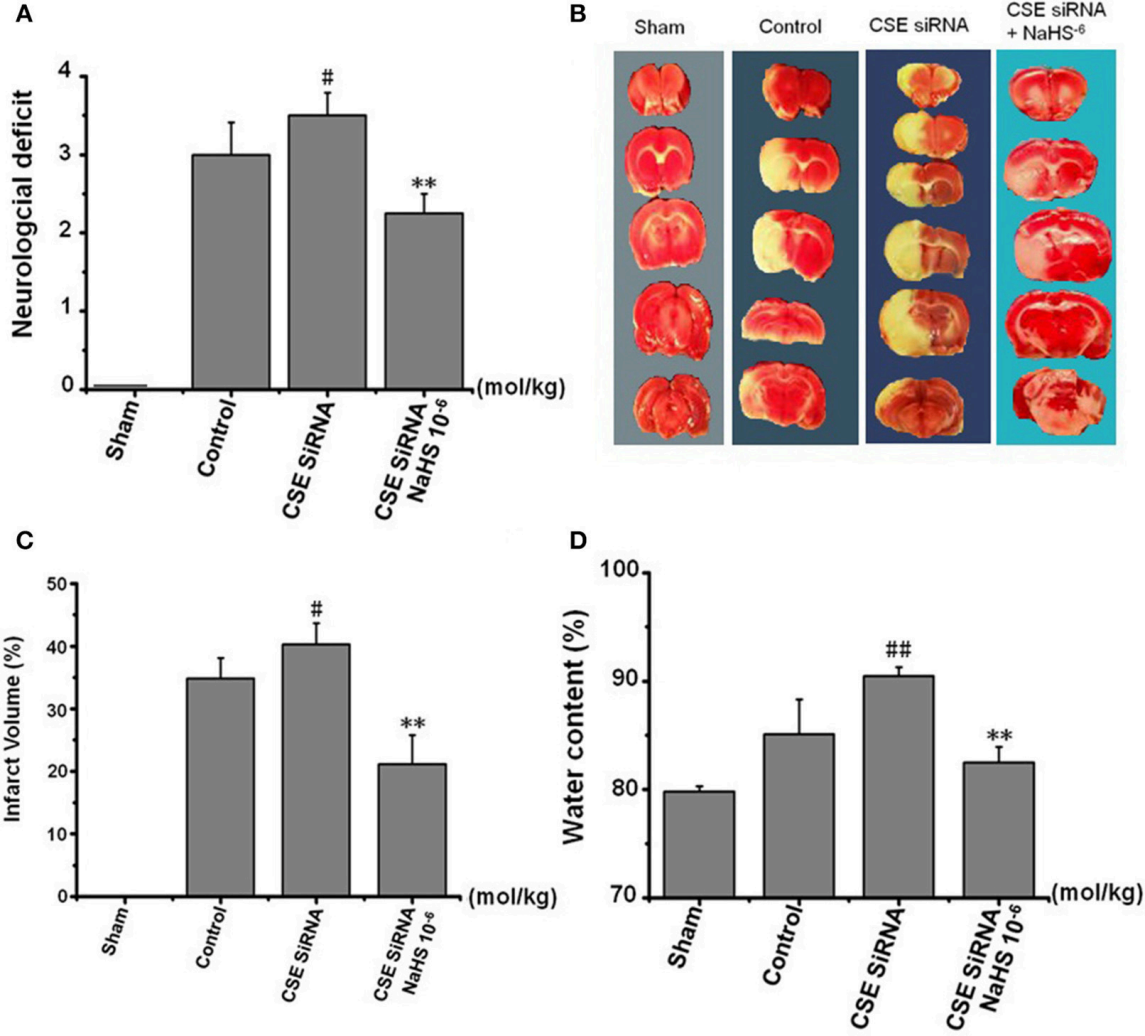
Effect of CSE knockdown (CSE siRNA) on neuronal injury induced by MCAO in rats (Mean ± SEM, *n* = 8). **(A)** Neurological deficits. **(B)** Infarct volume. **(C)** Quantitative analysis of brain infarct volume. **(D)** Brain water content. ^#^*P* < 0.05 vs. control, ^##^*P* < 0.01 vs. control, ^**^P < 0.01 vs. CSE siRNA.

The neurologic deficit scores of rats are presented in Figure 5A. No neurologic deficits were observed in the sham group. Moderate neurologic deficits (average score: 3) were observed at 24 h after reperfusion in control group rats, while in the CSE-siRNA group, the rats had significant neurologic deficits (average score: 3.5), and interestingly, the neurologic deficits were remarkably inhibited by 1 × 10^−6^ mol/kg NaHS supplementation within 30 min after ischemia.

### Effect of H_2_S on the infarction volume

As shown in Figures 5B,C, I/R remarkably induced cerebral infarction in rats. However, the increase of the infarction volume in the CSE knockdown rats was more significant than that in the control group rats. NaHS (1 × 10^−6^ mol/kg) supplementation significantly reduced the infarction volume in CSE knockdown rats alike.

### Effect of H_2_S on brain water content in rats

Brain water content among the other factors is regarded as being responsible for the neuronal dysfunction after brain ischemia (22) and can be used as an indicator of brain edema (21). The results (Figure 5D) showed that MCAO markedly increased the brain water content in CSE knockdown rats when compared to untreated rats of the control group, which could be significantly inhibited by NaHS (1 × 10^−6^ mol/kg) supplementation.

### Serum LDH activity and MDA level in brain tissue

**Figure 6:**
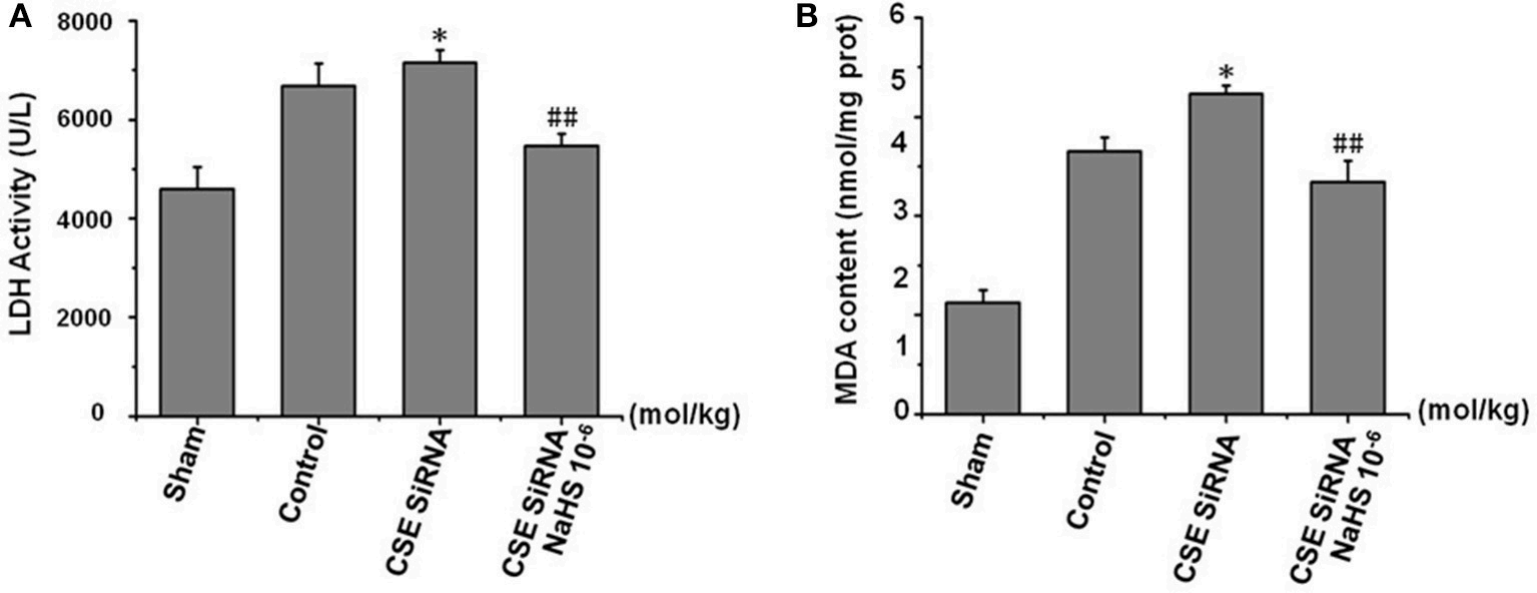
Effect of CSE knockdown (CSE siRNA) on MCAO rat serum LDH activity and MDA content in brain tissue (mean ± SEM, *n* = 8). **(A)** Serum LDH activity. **(B)** MDA content in brain tissue. ^*^P < 0.05 vs. control; ^##^*P* < 0.01 vs. CSE isRNA.

LDH leakage from cells to serum and MDA, a product of lipid peroxidation, are major indexes of ischemia injury. In the control group (Figure 6) there was a significant increase of LDH activity in serum and MDA content in cerebral tissue induced by cerebral I/R, and the results indicated that I/R could induce significant cerebral injury. However, the injury was more remarkable in CSE knockdown rats than in the control group (*p* < 0.01) and was significantly inhibited by NaHS (1 × 10^−6^ mol/kg) supplementation.

These results confirmed that H_2_S has a remarkable protective effect on MCAO-induced neuronal injury.

## Discussion

Cerebral I/R injury is a serious and common clinical disease. The tissue plasminogen activator (t-PA) is the only FDA-approved treatment for acute brain ischemia. However, only a small proportion of brain ischemia patients are eligible to receive tPA treatment because it carries a high risk of secondary impairments, such as bleeding/hemorrhagic transformation and severe neurodegeneration (23, 24). Thus, the main priority is to explore the neuroprotective strategies and find new drugs for possible clinical use.

Previous studies have reported that cerebral I/R decreases endothelial vasoreactivity, impairs blood flow restoration, and also causes further brain injury (5). H_2_S is a novel vasoactivator, and research has pointed out that H_2_S is helpful for cerebral ischemic injury. Although the role of H_2_S on cerebral I/R injury has attracted the interest of many researchers, the mechanism involved in the effect of H_2_S on cerebral I/R injury is still not completely clear (9). The aim of this study was designed to clarify the vascular protection of H_2_S on neurovascular dysfunction after cerebral I/R. The results showed that the contraction and dilation of MCA profoundly decrease after cerebral I/R. The reduction in the contraction and dilation was significantly ameliorated by 1 × 10^−5^ ~ 1 × 10^−7^ mol/kg NaHS supplement. Not surprisingly, in CSE knockdown rats, MCA almost loses its dilation to ACh or vinpocetion, and constriction to U46619 after cerebral I/R, which could also be significantly ameliorated by 1 × 10^−6^ mol/kg NaHS supplement. These data indicated that H_2_S had a significant protection on cerebrovascular dysfunction induced by cerebral I/R.

We next sought to investigate the mechanism of MCA relaxation on to H_2_S using K_Ca_ channel blockers and endothelial removal. As has previously been established, H_2_S has been classified as a novel gasotransmitter signaling molecule in CNS, which is involved in various signal transmissions such as the regulation of ion channels (14). Furthermore, the cerebral endothelium has a key role in the regulation of vascular tone because the endothelium could release H_2_S and other relaxing factors such as NO and PGI_2_ to relax vascular smooth muscle cells (VSMC) (17). In the present study, we found that the relaxation of isolated MCA to H_2_S donor NaHS and L-Cys (substrate of endogenous H_2_S-producing enzyme) was abolished by the co-application of the intermediate-conductance K_Ca_ channel blocker CTX and small-conductance K_Ca_ channel blocker Apa. In parallel, similar dilation of MCA elicited by L-Cys was blocked by endothelial removal. However, adding endothelium cells (EAhy926) cultured *in vitro* to luminal perfusate could mediate non-NO and non-PGI_2_ vasorelaxation to ACh in endothelium-denuded MCA, but the relaxation was abolished by CSE knockdown in EAhy926 cells. These results provided solid evidence that the vasodilation of cerebral vessels to H_2_S is endothelium-dependent and might relate to activate the K_Ca_ channel.

To further confirm the protective effects of H_2_S on neuronal injury after cerebral I/R, the MCAO was still used as a model of focal cerebral I/R and associated with an increase of infarction volume, brain water content, and neurological scoring (25). Our data revealed that cerebral I/R injury led to a significant increase of cerebral infarction, brain edema, and neurological deficits, thereby suggesting an eminently neuronal injury. In addition, it is widely accepted that oxygen-free radicals in neurocytes induced by cerebral I/R injury and subsequent lipid peroxidation play a key role in the pathophysiology of I/R injury. Hence, like MDA, a product of lipid peroxidation, LDH leakage has also been applied to assess cerebral I/R injury (9, 26). In agreement with the previous result, we found that cerebral I/R injury led to a significant increase of serum LDH activity and MDA content in MCAO rats. However, all the above injury indicators occurred more grievously in CSE knockdown rats than in the normal control group, and could be remarkably inhibited by 1 × 10^−6^ mol/kg NaHS supplementation. Together with treatment, the effect of H_2_S donor NaHS on the injury suggests that H_2_S could inhibit cerebral I/R-induced increases of cerebral infarction, brain edema and neurological deficits, LDH leakage, and lipid peroxidation. These findings provide more details and demonstrate that H_2_S has a protective effect on neuronal injury induced by MCAO.

In conclusion, our study is the first to show the multifaceted vasoprotection of H_2_S on cerebral I/R injury. We found that (1) both endogenous and exogenous H_2_S had eminent protection on vasomotor dysfunction induced by MCAO in rats; (2) K_Ca_ channel might be involved in the cerebrovascular relaxation to H_2_S; (3) the cerebrovascular relaxation to H_2_S is endothelium-dependent; (4) both endogenous and exogenous H_2_S had a protective effect on neuronal injury after cerebral I/R in rats.

## Author contributions

Z-WC, J-YW, and MW participated in research design and experiments. H-HJ and X-JS contributed new reagents and analytical tools. H-HJ and X-JS performed data analysis. Z-WC, J-YW, and Y-NL contributed to writing of the manuscript.

### Conflict of interest statement

The authors declare that the research was conducted in the absence of any commercial or financial relationships that could be construed as a potential conflict of interest.
